# Canadian Veterans’ Experiences of Living with Chronic Pain: A Descriptive Qualitative Study

**DOI:** 10.1080/24740527.2024.2361006

**Published:** 2024-06-10

**Authors:** Moizza Zia Ul Haq, Vahid Ashoorion, Cheng En Xi, Eileen Wang, Natasha Ross, Nandana Parakh, Jason W. Busse, Andrea J. Darzi, Elizabeth Alvarez

**Affiliations:** a Department of Health Research Methods, Evidence and Impact, McMaster University, Hamilton, Ontario, Canada; b Department of Anesthesia, McMaster University, Hamilton, Ontario, Canada; c Faculty of Health Sciences, McMaster University, Hamilton, Ontario, Canada; d Global Health, Faculty of Health Sciences, McMaster University, Hamilton, Ontario, Canada; e Michael G. DeGroote Centre for Medicinal Cannabis Research, McMaster University, Hamilton, Ontario, Canada; f Centre for Health Economics and Policy Analysis (CHEPA), McMaster University, Hamilton, Ontario, Canada

**Keywords:** veterans, chronic pain, Canadian Armed Forces, Veterans Affairs Canada, health services, domains of well-being

## Abstract

**Background:**

An estimated 30% of veterans live with chronic pain, compared to 20% of Canadians in the general population. Veterans face health care challenges upon release from the military, increasing difficulties in obtaining chronic pain care.

**Aims:**

We explored experiences of Canadian Armed Forces veterans living with chronic pain, their transition from military to civilian care, perceived barriers and facilitators to chronic pain care, and impacts of their pain on the domains of well-being.

**Methods:**

We conducted a qualitative descriptive study using semistructured interviews. We used a deductive/inductive approach to derive themes and concepts from interview transcripts.

**Results:**

Thirty-five veterans living with chronic pain participated. Participants reported that pain affected their lives in numerous ways, including negatively impacting relationships and limiting activities of daily living and leisure. They identified barriers to care, including lack of access to family doctors or health care services, reluctance to ask for help, and challenges in obtaining coverage for services from Veterans Affairs Canada. Facilitators included support from other veterans and online resources. Chronic pain had bidirectional effects on domains of well-being.

**Conclusions:**

Experiences of pain varied among Canadian veterans, and military culture played a role in perceptions and management of pain. Barriers and facilitators to chronic pain care were highlighted from their time in the military into their transition to civilian care. Participants described the impact of chronic pain on their overall well-being. Determining whether these findings are relevant to a larger population of Canadian veterans will be important for future research and knowledge translation to improve chronic pain care for Canadian veterans.

## RÉSUMÉ

**Contexte :** On estime que 30 % des anciens combattants souffrent de douleur chronique, contre 20 % des Canadiens dans la population générale Les vétérans sont confrontés à des défis en matière de soins de santé lorsqu'ils quittent l'armée, ce qui augmente les difficultés pour obtenir des soins pour la douleur chronique.

**Objectifs :** Nous avons exploré les expériences des vétérans des Forces armées canadiennes vivant avec une douleur chronique, leur transition des soins militaires aux soins civils, les obstacles et les facilitateurs perçus en matière de soins pour la douleur chronique, ainsi que les effets de cette douleur sur les différents aspects de leur bien-être.

**Méthodes :** Nous avons réalisé une étude qualitative descriptive en utilisant des entretiens semi-structurés. Une approche à la fois déductive et inductive a été utilisée pour extraire des thèmes et des concepts à partir des transcriptions des entretiens.

**Résultats :** Trente-cinq anciens combattants souffrant de douleur chronique ont participé à l'étude. Les participants ont déclaré que la douleur affectait leur vie de nombreuses façons, notamment en ayant un impact négatif sur leurs relations en limitant les activités de la vie quotidienne ainsi que les loisirs. Ils ont recensé des obstacles aux soins, notamment le manque d'accès à des médecins de famille ou aux services de soins de santé, la réticence à demander de l'aide, et les difficultés à obtenir une couverture pour les services d'Anciens Combattants Canada. Les facilitateurs comprennent le soutien d'autres anciens combattants et les ressources en ligne. La douleur chronique a eu des effets bidirectionnels sur différents aspects de leur bien-être.

**Conclusions :** Les expériences de la douleur varient parmi les anciens combattants canadiens, et la culture militaire joue un rôle dans les perceptions et la prise en charge de la douleur. Les obstacles aux soins pour la douleur chronique, ainsi que les facilitateurs, ont été mis en évidence depuis leur temps dans l'armée jusqu'à leur transition vers les soins civils. Les participants ont décrit l'effet de la douleur chronique sur leur bien-être général. Il sera important de déterminer si ces résultats sont pertinents pour une population plus large d'anciens combattants canadiens dans le cadre de recherches futures et de l'application des connaissances, afin d'améliorer les soins pour la douleur chronique chez les anciens combattants canadiens.

## Background

An estimated 30% of veterans live with chronic pain compared to 20% of the general Canadian population.^1^ Women veterans, who comprise 16% of veterans,^2^ report higher rates of chronic pain compared to men,^3^ and more than 85% of veterans with mental illness also experience chronic pain.^4,5^ A Veteran is defined as a former member of the Canadian Armed Forces (CAF) who successfully underwent basic training and was honorably discharged, and there are currently approximately 461,240 veterans in Canada.^6^ Veterans Affairs Canada (VAC), which is responsible for pensions, benefits, and services for veterans in Canada, reported several impacts of chronic pain in Canadian veterans, including interference with work, life stress, activity limitations, and suicidal ideation.^7^

Canadian veterans may face several health care challenges upon release from their military service, as they transition from a federally run, highly specialized health care system in the military to a community-based, civilian health care system.^8^ Additionally, though veterans in Canada may be eligible for additional health benefits through VAC, only 35% of veterans report receiving benefits.^8^ Studies in the United States found that veterans identified several barriers to uptake of various pain treatment modalities, including transportation, costs, and limited insurance coverage for services. Facilitators to engaging in treatment included having services nearby and having a variety of different treatments available.^9,10^

Further, military culture tends to downplay the experience of pain; for instance, in the military, pain is often regarded as “something to be endured and not discussed” (200) and, as a result, veterans may find it difficult to discuss or seek help for chronic pain.^11^ Furthermore, the military may foster attitudes such as self-reliance and the need to “just [get] on with it,” which may impact how veterans cope with and manage chronic pain.^12^

The research and policy directorates at VAC developed seven domains of Veteran well-being through a consensus-seeking, multidisciplinary process informed by published literature, expert consultations, and evidence from the Life After Service Studies.^13,14^ This framework was developed to support the design of policies and programs for veterans’ well-being during and after the military-to-civilian transition and includes (1) health, (2) employment and meaningful activity, (3) finances, (4) social integration, (5) life skills and (6) housing and physical environment, and (7) cultural and social environment.^13,14^ However, how chronic pain affects these domains or how aspects of care in these domains affect chronic pain in Canadian veterans is not known. Therefore, it is vital to understand the experiences of veterans living with chronic pain (VLwCP), barriers and facilitators to care, and relationships with the domains of well-being to inform the planning and provision of relevant services.

Thus, our study aimed to explore the experiences of Canadian Armed Forces VLwCP, their transition from military to civilian care, perceived barriers and facilitators to chronic pain care, and the impact of their pain on the seven domains of well-being, which are health, employment and meaningful activity, finances, social integration, life skills and housing and physical environment, and cultural and social environment.

## Methods

### Study Design

We conducted a descriptive qualitative research study with Canadian VLwCP. We chose a descriptive approach because it was appropriate to gain insight on and describe participants’ experiences in easily understood language and from their perspectives.^15^ We used in-depth interviews and thematic analysis, which involved identifying, analyzing, and reporting themes within the data.^16^ We followed the Consolidated Criteria for Reporting Qualitative Research checklist.^17^

### Participants

We recruited English- or French-speaking veterans of the CAF aged 18 years or older living with chronic pain. We utilized Health Canada’s definition of chronic pain, where chronic pain is defined as pain that continues for 3 months or longer.^18^ We utilized VAC’s definition of veteran, where a Veteran is defined as a former member of the CAF who successfully underwent basic training and was honorably discharged.^6^

### Sampling and Recruitment

We used three sampling strategies to identify participants: (1) intensity sampling for CAF VLwCP, (2) snowball sampling to identify other VLwCP, and (3) maximum variation sampling to capture a range of viewpoints, including men and women, having served in different branches of the CAF, diverse racial or ethnic backgrounds, and geographical distribution.

Participants were recruited through Canada-wide networks maintained by the Michael G. DeGroote National Pain Center at McMaster University and the Chronic Pain Center of Excellence for Canadian Veterans. We also posted social media advertisements to participate in our study on Twitter, Facebook, Instagram, and LinkedIn. Additionally, we reached out to organizations and clinics across Canada that served veterans with chronic pain to distribute our recruitment messages. Veterans were invited to contact a research assistant (V.A.) via e-mail or phone, who then determined eligibility and sent interested participants a letter of information to inform them about the nature of the study, their rights as study participants, potential risks, confidentiality of their data, voluntary entry into the study, and their ability to withdraw from the study at any time. Prior to enrollment, written informed consent was obtained from all veterans. Participants were compensated with a $30 online gift card.

### Data Collection

Enrolled participants were asked to complete a demographic questionnaire and then scheduled for an interview with a research assistant. Our interview guide asked about veterans’ experiences living with chronic pain, the transition from military to civilian care, facilitators and barriers to chronic pain care, and the impact of their pain on the seven domains of well-being.

The demographic questionnaire (Supplemental Material 1) and the interview guide (Supplemental Material 2) were reviewed by two Canadian VLwCP who were not interviewed. One of five trained research assistants (V.A., M.U., C.X., N.R., E.W.) conducted a semistructured in-depth qualitative interview (expected to last 45–90 min) with each participant between August 2022 and January 2023. Prior to recruitment, interviewers were trained in research ethics and reflexivity in qualitative research and received training from a Veteran expert on Veteran identity and integration. Interviewers also conducted mock interviews with one another to ensure consistency in interview styles and the use of neutral language. Interviews were conducted through video conference (Zoom) or over the phone, as preferred by each participant.

Interviews were audio recorded and the research assistant also took field notes during each interview. Recordings from the interviews were downloaded onto the research assistant’s laptop and then uploaded onto a shared password-protected and encrypted storage folder (McMaster University’s SharePoint). (NVivo Version 14. 2023. www.lumivero.com) NVivo software was used for initial transcription. Deidentified transcripts were uploaded to SharePoint. We gave all participants a study ID number, and their data were kept separately from the study key to ensure confidentiality. We collected data until we achieved saturation, the point at which no new substantive information was obtained from participants.

### Data Analysis

Two researchers independently coded each transcript in NVivo following a deductive/inductive approach.^19^ The initial codebook was informed by the interview guide and other codes were added as guided by the data. Through an iterative process, we grouped together the codes to develop concepts and themes that emerged from the data. We met on a weekly basis to review and discuss emerging concepts and themes. To promote trustworthiness of our findings, we employed member-checking. Participants were asked during the informed consent process and in the interview whether they agreed to provide feedback on our preliminary findings; among those, we selected a variety of participants based on gender, geographic location, and branch of the CAF in which they served. Given the amount of results gathered and to respect participants’ time, we divided the results by half and sent each half to three participants for member-checking. Five of six participants responded. Participants were asked the following questions: Is there anything you feel is not accurate? Is there anything you feel is not relevant? Is there anything missing? Participants provided written feedback through e-mail, and we incorporated suggestions upon team discussion. One VLwCP who did not participate in the study also provided an external review of our findings. Given the limited number of suggested changes, we did not pursue further member-checking. We also kept an audit trail of our data analysis.

### Researcher Reflexivity

Each team member who conducted interviews completed a reflexive exercise to understand their own positionality, biases, and predetermined ideas (Supplemental Material 3).^17^ We continuously discussed new learnings and how our biases and assumptions may have affected or been affected by data collection and analysis.

### Ethics

This study was approved by the Hamilton Integrated Research Ethics Board, Project #14847, and complies with the Declaration of Helsinki.

## Results

We conducted 35 interviews that lasted an average of 79 min (range = 43 to 178 min). Participants ranged in age from 26 to 75 years, 69% (*n* = 24) were men, and they were evenly distributed across the Central, Prairie, West Coast, and Atlantic regions of Canada (Table 1). Most participants (89%, *n* = 31) identified as White/European, with three individuals identifying as Asian, Black, or Indigenous. Most veterans were married or common law (74%, *n* = 26).

**Table 1.** Demographic characteristics of participants (*n* = 35).

Characteristic	Number (%) of participants
Age	
<35	1 (3)
36–45	6 (17)
46–55	13 (37)
56–65	12 (34)
66–75	3 (9)
Gender	
Male	24 (69)
Female	11 (31)
Race or ethnicity	
White/European	31 (89)
Asian	1 (3)
Black	1 (3)
Indigenous	1 (3)
Prefer not to answer	1 (3)
Region of Canada^a^	
Central	10 (29)
Prairie provinces	9 (26)
West coast	8 (23)
Atlantic	8 (23)
Region of residence	
Urban	14 (40)
Suburban	12 (34)
Rural	9 (26)
Relationship status	
Married	25 (71)
Divorced	5 (14)
Single/never married	2 (6)
Common law	1 (3)
Separated	1 (3)
Widowed	1 (3)
Current household gross income	
$25,000 to $49,999	3 (9)
$50,000 to $74,999	6 (17)
$75,000 to $99,999	8 (23)
$100,000 to $150,000	12 (34)
More than $150,000	5 (14)
Prefer not to answer	1 (3)

^a^Atlantic (Newfoundland and Labrador, Prince Edward Island, Nova Scotia, New Brunswick), Central (Quebec, Ontario), Prairie (Manitoba, Saskatchewan, Alberta), West Coast (British Columbia).

Most veterans had completed high school (29%, *n* = 10) or had a college or university degree (66%, *n* = 10), and most had served in the CAF for more than 23 years (69%, *n* = 24; Table 2). Most participants had served in the Army (46%, *n* = 16), followed by the Air Force (23%, *n* = 8) and the Navy (17%, *n* = 6). Most had been out of the CAF for more than 10 years (53%, *n* = 18), and, at the time of interview, 60% were retired (*n* = 21) and 17% were unemployed (*n* = 6). The majority (77%, *n* = 27) had lived with chronic pain for more than 10 years, and 94% (*n* = 33) experienced pain daily (Table 3). Most veterans (60%, *n* = 21) reported mixed types of chronic pain, and 80% (*n* = 28) reported a chronic pain condition covered by VAC.

**Table 2.** Employment, education, and military-related characteristics of participants (*n* = 35).

Characteristic	Number (%) of participants
Employment status	
Retired	21 (60)
Unemployed	6 (17)
Employed, full-time	4 (11)
Employed, part-time	1 (3)
Other	3 (9)
Highest formal education level	
High school	10 (29)
College degree	10 (29)
University degree	13 (37)
Other	2 (6)
Branch of the Canadian Armed Forces	
Army	16 (46)
Air Force	8 (23)
Navy	6 (17)
Multiple branches	4 (11)
Royal Canadian Mounted Police	1 (3)
Length of time served in the Canadian Armed Forces	
2–9 years	4 (11)
10–19 years	7 (20)
>20 years	24 (69)
Length of time out of or retired from the Canadian Armed Forces (*n* = 34)	
<2 years	3 (9)
2–9 years	13 (38)
10–19 years	14 (41)
>20 years	4 (12)

**Table 3.** Chronic pain characteristics of participants (*n* = 35).

Participant chronic pain characteristics	Number (%) of participants
Length of time living with chronic pain	
1–5 years	3 (9)
6–10 years	5 (14)
>10 years	27 (77)
Type of chronic pain	
Nociceptive	6 (17)
Neuropathic	3 (9)
Nociplastic	4 (11)
Mixed type	21 (60)
Uncertain	1 (3)
Frequency of pain	
Daily	33 (94)
Weekly	2 (6)
Have chronic pain condition(s) approved byVeterans Affairs Canada	
Yes	28 (80)
No	7 (20)
If do not have a chronic pain condition approved by VAC, have submitted a claim to VAC for achronic pain problem(s) (*n* = 7)	
No	6 (86)
Yes	1 (14)

The following sections describe participants’ causes of chronic pain, effects of chronic pain on veterans’ lives, influences of military culture on chronic pain, programs and services used for chronic pain, barriers and facilitators to obtaining services for chronic pain by stages in the transition process, and relationships between chronic pain and the seven domains of well-being.

### Causes of Chronic Pain

Mostly, veterans attributed their pain to cumulative injuries or chronic wear and tear and in some cases to singular events, such as an accident or fall. While serving, their pain was often exacerbated by continuing to push themselves physically or causing re-injury. Some participants acknowledged not reporting injuries so they could be deployed.

You block [the pain] out. You just take medication and you block it out, because it’s all about the mission, it’s all about going on deployments. (1053)

Other factors that contributed to injuries, chronic wear and tear, or chronic pain were (1) lack of or ill-fitting gear during trainings or in the field because military equipment was made for certain body types or not customized to women; (2) keeping up with training standards expected of younger and bigger men; (3) rough rides on ships and aircrafts; (4) for small aircraft crew, having their heads down with excess weight from their helmet, causing neck and back issues; and (5) for one participant, sun exposure without protection caused skin cancer with the need for surgery and resulting neuropathic pain. Several participants specifically highlighted the rucksack march (training exercise involving walking a distance while carrying a weight in a backpack or rucksack)^20^ and fireman carry (a lifting technique allowing one person to carry another person by placing the individual across their shoulders)^21^ as times of injury, when expected to carry heavy loads. Even though injuries typically occurred when participants were younger, they noted that pain had increased or was expected to increase with age.

### Effects of Chronic Pain on Veterans’ Lives

Participants identified several aspects of their lives that were impacted by their chronic pain. Participants noted that pain limited their activities of daily living, including grooming, dressing, toileting, transferring or ambulating, and eating. For some, it necessitated using aids such as walkers, wheelchairs, and/or canes. Some expressed feelings of disappointment or humiliation at not being able to do these tasks. Participants also noted that chronic pain limited other day-to-day activities including household duties, driving, grocery shopping, and leisure activities such as playing sports. A few expressed that they would still participate in such activities but were in pain when doing so. Some participants stated that this shift from being active to more sedentary due to their pain was psychologically challenging. Furthermore, some participants noted that pain limited their ability to work due to physical requirements of their job. Some could no longer work, and others needed accommodations or would hide their pain at work. Participants highlighted that they had to base their ability to work on their worst day, rather than their best, in relation to their pain.

I’ve tried to, I guess, mitigate or modify my activities in order to keep doing some or most of the things, but the reality is it either prevents, limits the activity, or it significantly restricts it. (1004)

Like some days, I can work out for 20 to 30 minutes … but there are days when I can’t bend over to put my socks or my shoes anymore. That’s pretty damn humiliating. (1005)

Participants were asked about the effect of pain on their relationships. Many participants noted that old connections were lost or new ones were difficult to form. Reasons included feeling like “I wasn’t who I was anymore” (1000), inability to do activities and socialize due to pain or anxiety, being in a “bad mood” (1033) or “irritable” (1048) due to pain, focus on pain annoying others, poor memory or concentration due to pain, or being embarrassed by their physical limitations. Some participants explained that “pain [was] very selfish” (1054) because it caused them to withdraw within themselves and not engage with others. Participants also felt that pain affected how others saw them, because people did not understand or did not always believe them. Other participants felt they became invisible and that only other veterans could understand them.

Being in so much pain makes it hard to be around people that don’t understand pain, and family is a big one on that, and some friends … because a lot of people don’t believe you’re in pain when they don’t see you with a crutch. (1055)

Some participants noted changes in caregiver roles with their children, spouse, or other family, which were difficult for both parties by adding responsibilities for others and causing feelings of guilt for participants. Some veterans noted that it was hard to engage in parenting, which impacted short- and long-term relationships with their children. At times, pain impacted intimacy in addition to increased irritability and limited social interactions, which could affect relationships with spouses or partners.

A few participants noted that it was difficult to accept their new reality of living with chronic pain and possibly not getting better, and some questioned their sense of value, especially if they could not go back to work. For many, pain affected all aspects of their life, decreased their quality of life, and consumed a lot of daily energy.

### Influences of Military Culture on Chronic Pain

Participants described several ways in which military culture impacted their pain. Some noted that the military attracted people with certain personality types, including people who were task-oriented, perfectionistic, and concerned about others or those who had family members who had been in the military. Veterans reported that when in the military, they felt they could not refuse orders. They discussed the military culture as being mission-centric with an obligation to duty. Because lives were at stake, participants felt they had to accept pain as part of the job or as a part of life.

“Pain is just a symbol that you’re still alive and pain is just leaving the body and making sure that you’re still alive.” So my tolerance for pain is high, because that’s the way we were trained. The biggest one they say this is just “pain is just weakness leaving the body.” (1033)

I was in the infantry, so it’s combat arms, that’s frontline troops—we don’t talk about pain, we accept pain is part of what we do. And it’s like, you know, jet lag for a pilot. It’s just going to go with what we do. You’re using your body. You’re doing a job. (1076)

Accepting pain was also important because if they could not function, they could negatively impact their team, miss out on important experiences or opportunities, or lose their military career. Some participants discussed a competitive environment in the military that was fostered by ideas of masculinity that encouraged being “harder, faster, and stronger than everyone” (1005), being an “alpha male” (1052), and having to “be a man” (1009) or “man up” (1048). One participant described this as a culture of “toxic masculinity” (1005), applying to both men and women. Furthermore, the military mentality of pushing through pain and not talking about it or seeking care caused members to work while in pain, which exacerbated injuries and led to further pain. Some participants felt that this was understandable when in the field but that it would have been helpful to have time to heal after injuries or intense trainings.

If I’m not physically fit, I didn’t have a job, so I would continue to work. Even though I was in pain, and I continue[d], it continually got worse. … And there was a [stigma], too, in the military where if you said you were hurting, that meant you were, like, sick, lame, or lazy or, you know, “You don’t want to go to sea or you don’t want to do that job, oh, you’ve got a sore knee. Boo hoo.” (1074)

For several participants, the mission-centric mindset translated to civilian life attitudes, where participants continued to push through activities or felt guilty or embarrassed if they could not perform. However, one participant noted that because of their military service, they were believed when they said they were experiencing debilitating pain.

Military culture also affected pain management, because they did not speak about pain, did not seek care unless it was dire, and/or self-medicated. Participants felt that military care offered band-aid and short-term pain treatment and there was no privacy of mental or physical health problems. A medical record could mean not being allowed to be deployed or do certain jobs, and, as a result, they did not report injuries, which led to difficulties with VAC claims when out of the military. For some, however, military culture helped them deal with pain, through enforcing goal setting, structure, and organization, which was useful in pain management. Some participants noted that the different branches varied in the pressure to not talk about or deal with pain or other issues.

Participants also mentioned that it was hard to talk with civilians about their experiences and they connected better with other veterans. One participant mentioned that because military life required moving often, it was difficult to create relationships outside the military, leaving few supports as a veteran. Some participants highlighted added hardships as women in the military, including encountering sexual harassment or dealing with sexual trauma and their pain not being taken seriously.

### Programs and Services Used for Chronic Pain

Veterans reported a considerable range of interventions they had received or were currently receiving (Appendix 1). Many participants described a lengthy journey of trialling many treatments over a period of years before arriving at a therapeutic regime that was helpful. Interventions that were currently used by the majority of veterans were stretching, massage therapy, exercise therapy, and cannabis. Psychotherapy was noted by several participants as helpful for developing coping strategies. One participant mentioned the need for support programs for spouses and children of veterans with chronic pain to help them understand and work together.

I regularly utilize massage therapy and chiropractic care to manage [the pain]. It’s not maintenance, it’s not acute stuff, it’s if I don’t see those professionals, I’ll decompensate even more. (1004)

### Barriers and Facilitators to Obtaining Services for Chronic Pain by Stages in the Transition Process

Participants described several barriers and facilitators at different stages in the process of obtaining services for chronic pain as veterans (Appendix 2). These stages included seeking health care during their time in service; transitioning from CAF to VAC care; identifying available services, programs, or benefits; applying for VAC coverage for services; and accessing and receiving services. Participants also highlighted that every Veteran had unique circumstances, so experiences of pain and access to services differed. Findings are presented in the order of stages as barriers and facilitators were specific to different stages.

While serving in the military, veterans noted that acute care was prioritized with limited attention to management of chronic complaints. Some services were difficult to access (e.g., massage, acupuncture, chiropractic), and there was poor coordination between health care services. For some, health care that was provided within the CAF was seen as timely and well coordinated.

[The military doesn’t] offer any other modalities that you have in civilian world. You couldn’t go to acupuncture, you couldn’t have osteopathy, couldn’t have registered massage therapy (RMT). So, while you were serving in the military, chronic pain isn’t talked about the military. (1054)

The doctor sees you right away in the military. … You get reminded about needing vaccinations, so you don’t even have to think about it. It’s looked after for you. Everything. You don’t have to advocate for anything. (1076)

A number of participants noted that they did not want to leave the military but could no longer do the job due to their pain. Participants also highlighted that during the transition, getting the right programs and supports in place quickly was important to do well both physically and mentally. Experiences transitioning from military to civilian life varied based on prior experiences integrating with civilian life, CAF branches, and being a reservist versus in the regular force. Military to civilian transition presented challenges due to the need to transfer records to civilian health care providers (HCPs). Veterans sometimes did not have supports to help with their chronic pain or were uncomfortable reaching out for help and were often unaware of what services were available to them. VAC case managers were noted to be variable, with some providing excellent support (including providing knowledge on what services to apply for and helping complete paperwork), whereas others were seen as barriers to care. Both the Military Family Resource Center and the Transition Center were endorsed by veterans as helpful for identifying support for chronic pain after release from the military.

I’ve had some case managers that have been excellent. … I had some very kind and compassionate case managers that were able to help me walk through what was going on. (1011)

Identification of civilian services for chronic pain care (including available services and providers, as well as eligible benefits and coverage) was seen as challenging, and the online MyVAC (https://www.veterans.gc.ca/eng/e_services) was seen as a helpful resource in this regard. Help from other veterans in finding chronic pain resources, including through Soldier ON (https://www.soldieron.ca/), Operational Stress Injury Social Support, and Facebook groups were also acknowledged as useful facilitators.

When it came to applying for coverage, participants highlighted that VAC was not a care provider and likened it to an insurance company that required evidence of pain in relation to their military service for services to be covered. Blue Cross handled VAC coverage. Many participants found that it was difficult and onerous to obtain coverage and identified a number of barriers, including having to prove the connection between their pain and military service, having to complete extensive paperwork, facing long wait times for approval, and, for some, being denied coverage. Having support from HCPs, family and friends, and other veterans was seen as a facilitator in this stage. Importantly, not all participants had problems obtaining coverage from VAC; some expressed that the services they wanted or needed were covered, and some found VAC to be very helpful.

The paperwork and the administration … is an arduous, long, painful administrative process which would discourage so many people from even going forward, which it did with me. I was so physically hurt and in so much pain when I was first injured in the military that, honestly, even doing that paperwork for me was extremely difficult and painful. (1054)

I didn’t know about Veterans Affairs and what they had to offer veterans, until maybe about … four years ago. So up until that point, I was doing everything on my own, and … I was in constant pain because I didn’t know how to manage it. … But when I found out about Veterans Affairs, they really took an interest, I mean, they’ve bent over backwards to help me with all my issues. I’m definitely in a far better spot now. (1068)

At the stage of accessing and receiving health care services for chronic pain, many participants expressed difficulty finding a family doctor, which resulted in not having someone to sign off on paperwork, make referrals, or help navigate the health care system. Furthermore, certain services could be unavailable or far distances away, particularly in small provinces or rural settings. Facilitators identified at this stage included having access to HCPs, especially those who were supportive of and understood veterans and VAC.

And with the chronic pain, due to the extent of my injuries, I’m supposed to have somebody following me just … to make sure that that is getting better and not getting worse. So it hinders some things because there’s a lot of things that I need to get doctors to sign off for VAC and because don’t have a doctor, it makes it hard. (1026)

Living where I do, I don’t really have access to pain clinics or, you know, follow-up care from pain clinics. (1043)

Further barriers and facilitators, as well as participants’ recommendations for improvement throughout this process, are described in Appendix 2.

### Relationships between Chronic Pain and the Seven Domains of Well-being

Participants were asked about the seven domains for measuring well-being in veterans (health, employment or other meaningful activity, finance, social integration, life skills, housing and physical environment, and culture and social environment). We asked about each domain’s relationships with chronic pain, which are described in Table 4. Services and supports used to address each domain and barriers, facilitators, and recommendations to improve each domain are described in Appendix 3 and are briefly highlighted below.

**Table 4.** The seven domains of Veteran well-being and their relationships with chronic pain.

**Health**
Keeping physically active can help with pain and improve mental health.
Chronic pain can be impacted by other health conditions.
Pain adds to mental stress or exacerbates mental health problems through lack of sleep, change in mood, limiting what you can or want to do, causing worry.
Pain increases with poor mental health: through unhealthy behaviors (including inactivity and not eating well), deconditioning and weight gain, worsened pain, isolation.
**Employment and meaningful activity**
Having a sense of purpose helps distract from the pain and keeps the body moving.
Not having a sense of purpose and being more sedentary is bad for chronic pain.
Work or other activities can exacerbate pain, cause flare-ups, or cause exhaustion.
Pain can make it difficult to work, find work, or be motivated.
Feeling like you should be working and not being able to affects mental health and well-being.
**Finances**
Can afford more services/treatments for chronic pain if financially well or with insurance coverage.
Do not have to work as much or in difficult jobs that exacerbate pain when financially secure.
Not being able to work or spending funds on chronic pain treatments affects finances.
Financial stress affects chronic pain, because stress aggravates pain and/or poor mental health.
**Social integration**
Being social is a helpful distraction from pain, but too much can feel overwhelming or worsen pain.
Having a social network of people is helpful for finding resources.
Stressful social situations worsen pain.
Feelings of isolation cause self-neglect, which leads to not attending treatments or not taking care of self, which can worsen physical health and pain.
Socializing is the first thing to go when in pain; managing pain allows one to venture out socially.
**Life skills**
Life skills help cope with pain and include seeking help, distracting from pain, learning activities/mindsets that help with pain, and avoiding activities that worsen pain.
Coping skills to deal with chronic pain and mental health are important for preventing suicides.
Certain types of coping to deal with pain such as alcohol and drug use cause harm.
**Housing and physical environment**
Housing considerations (e.g., elevators, single-floor setup, handrail) can help with mobility.
Housing maintenance (e.g., yard work, shoveling, mowing lawn) can increase pain.
Modifying house maintenance activities, having housing that requires less maintenance, or having maintenance taken care of or covered helps decrease or avoid pain.
Affordable housing may lead to long travel distances for services and can exacerbate pain.
Climate and temperature (e.g., dampness or cold, barometric pressure) can increase pain.
Environmental exposure and air quality can lead to decreased activity and increase in pain.
**Culture and social environment**
Mentality, including work ethic and fitness from being in the military, may help deal with pain.
Having to explain or dispel stereotypes or hide pain can be stressful and increase pain.

#### Health

Services to address health included primary care and specialists, physical supports for pain, and mental health services such as counseling, psychologists, psychiatrists, psychotherapists, and antidepressant medications. Barriers in this domain included not having a family doctor, having unsupportive HCPs, difficulty finding services, and lacking coverage by VAC. Facilitators included supportive HCPs, helpful case managers, and, for some, support from VAC.

#### Employment and Meaningful Activity

Services and supports for this domain, as identified by participants, included activities that provided a sense of purpose (including but not limited to employment); activities with other veterans, such as Soldier On events; and supports for retraining and job finding, including VAC’s Education and Training Benefit and Vocational Rehabilitation program. Barriers in this domain included difficulty in transitioning to the civilian workforce and, for some, not feeling well-supported by the Education and Training Benefit or the Vocational Rehabilitation program. Facilitators in this domain included receiving support from chronic pain or mental health providers and having supportive employers.

#### Finances

Veterans identified several financial supports and services, including their military pension, long-term disability, VAC benefits and coverage (including Income Replacement Benefit, pain and suffering benefits, critical injury benefits), and self-supports. Barriers in the financial domain included lack of information on available supports, difficult or long processes for receiving benefits or coverage, and insufficient coverage or benefits for current or future needs. Facilitators in this domain included military financial education opportunities and supportive case managers or service providers.

#### Social Integration

Supports and services to help with social integration included family supports, counseling and mental health supports, and organizations such as Project Trauma, the Royal Canadian Legion, and Soldier On. Barriers to social integration included personal difficulties in forming relationships and lost camaraderie following discharge from the military or after veterans’ events like the Invictus Games. Facilitators to social integration included having friends or family who checked in on them and provided help.

#### Life Skills

Services and supports that addressed life skills for veterans included mental health services and physical health supports for pain, such as occupational therapy. Barriers in this domain included lack of education on life skills from being the military, lack of information on available supports, and difficulty asking for help. Facilitators identified by some veterans included skills and discipline taught in the military, which helped with coping; prior education and experiences; and faith.

#### Housing and Physical Environment

Services and supports for housing and physical environment included VAC-offered occupational assessments, coverage for home modifications, and VAC’s Veterans Independence Program (VIP), which helped pay for services such as snow removal, house cleaning, and lawn care. Barriers in this domain included not being able to receive VAC coverage for some and VIP not being sufficient to cover services. Facilitators in this domain included chronic pain services that considered and addressed housing.

#### Culture and Social Environment

In the domain of culture and social environment, supports identified by veterans included involvement in civilian groups, as well as the Operational Stress Injury clinic. Barriers for veterans in this domain included feeling that their pain or mental health was invisible and feeling a lack of understanding of the experiences of veterans from the general population. Facilitators in this domain included receiving support and understanding from HCPs and being part of social groups. Some participants found that, in their experience, people were understanding of veterans and/or of people experiencing pain.

## Discussion

### Summary of Main Findings

This descriptive qualitative study included in-depth interviews of 35 CAF VLwCP to explore their experiences of living with chronic pain, their transition from military to civilian care, perceived barriers and facilitators to chronic pain care, and the impact of their pain on the seven domains of well-being. Participants mentioned that their chronic pain was caused by singular events or cumulative injuries and chronic wear and tear, and several factors could contribute to injury, including lack of military equipment for all body types, keeping up with training standards, rough rides on ships and aircrafts, carrying heavy military gear, and rucksack marches. Participants noted effects of pain on day-to-day activities, mental health, and social relationships. These included pain limiting their daily living activities, which could cause feelings of disappointment or humiliation; limiting their ability to work; and impacting their relationships with friends, spouses, and children. For many participants, pain affected every aspect of their abilities and decreased their quality of life. Many participants highlighted the relationships between pain and mental health through a cycle of pain, depression or low mood, and unhealthy behaviors (including inactivity and not eating well).

Participants described several ways in which military culture influenced their pain. First, the military attracted people who were task-oriented, dogged, perfectionistic, and concerned about others. Military culture also promoted a mission-centric mindset, which led to ignoring and pushing through with pain. Furthermore, participants noted having to accept pain as part of the job and not talking about pain, which limited treatment for it. Participants discussed programs, services, or medicines used for their chronic pain, with mixed reactions and results for most types of services. Participants described barriers and facilitators while seeking health care during their time in service: transitioning from CAF to VAC care; identifying available services, programs, or benefits; applying for VAC coverage for services; and accessing and receiving chronic pain services. Participants described the relationships between chronic pain and the seven domains for well-being and services to improve each domain.

### Alignment with Existing Literature

Findings from our study built on existing literature on veterans’ experiences of chronic pain, perceived barriers to accessing services and supports, and the role of military culture on chronic pain. A UK-based study found that VLwCP’s behaviors and emotions were shaped by their chronic pain, whereby their pain limited their ability to partake in activities such as running or playing with their children, which was coupled with emotional responses such as sadness or disappointment.^12^ Similarly, a qualitative study of American veterans revealed that women veterans living with chronic pain felt a major impact of pain on their quality of life, whereby the pain affected all aspects of lives, including physical, psychological, and social aspects.^22^ Another study of American veterans by Matthias et al. also described the emotional toll of chronic pain that included annoyance frustration, hopelessness, depression, and anger.^23^ Our study captured similar themes.

Veterans in the United Kingdom and United States expressed attitudes of “getting on with it” and pushing through the pain that were fostered by their time in the military, which was also expressed by veterans in our study.^12,22^ Furthermore, in a qualitative study by Kithulegoda et al.^24^ exploring challenges identified by Canadian VLwCP to identify priorities regarding chronic pain research, an emerging theme was the need to improve care for pain in the military, because the culture in the military was not conducive to reporting pain and provided little time to recover after injuries. Similarly, our study found that military culture limited discourse surrounding the experience of injuries and pain, impacting veterans’ ability to adequately report pain and manage it. Some participants discussed the impact of ideas of masculinity in the military, and one participant identified a culture of toxic masculinity in the military, which is understood as negative societal repercussions that relate to attitudes and behaviors that are expected of men.^25^ Similarly, Berdahl et al. describe a theory of “masculinity contest culture” that is fostered in organizations, including the military, whereby individuals must compete and prove one’s masculinity to others.^26^

Additionally, Williams et al.^27^ conducted a qualitative inquiry into the health and well-being of Canadian veterans during medical release. Both Kithulegoda et al. and Williams et al. noted several challenges in the transition from military to civilian life, including uncertainty and difficulty navigating the transition process, lack of support, and loss of identity or sense of purpose upon exiting the military.^24,27^ Our study captured similar themes and also highlighted facilitators in the transition process such as supportive case managers, family and community support, and the ability to prepare for the transition ahead of time.

Additionally, Williams et al.^27^ highlighted challenges in accessing health care for veterans, such as extensive paperwork and long wait times when applying for VAC benefits, and Kithulegoda et al.^24^ noted the challenges in accessing services through VAC. Our study identified similar barriers regarding the process of applying for and receiving coverage from VAC and also highlighted additional barriers including difficulty in navigating VAC, the need to provide proof of injury or pain during military service, denials of claims, and limited coverage. Limited coverage, lack of support from HCPs, and lack of information were also barriers faced by VLwCP in the United States.^9,10^ Our research also identified facilitators in this stage, including supportive medical professionals, nearby and available services, and support from providers and VAC.^9,10^

Williams et al. and Kithulegoda et al. discussed the lack of readiness during the transition, particularly in terms of finding a family doctor or specialists, experiences that were echoed in our study.^24,27^ Kithulegoda et al.^24^ also explored the importance of improving knowledge of pain management, and of military life among civilian HCPs, which was reflected in our study as well.

Williams et al.^27^ also discussed the difficulty faced by veterans in finding employment or managing and balancing employment and medical treatments. Our study reinforced this difficulty and extended further to capture other relationships between employment and chronic pain in that employment can help find purpose, but work or other activities can exacerbate pain or cause flare ups or exhaustion. Williams et al.^27^ also found relationships between physical and mental health, with participants describing a “vicious cycle” between the two. Participants in our study similarly highlighted the multiplicative effects of physical and mental health issues and described a cycle of mental stress and pain. Moreover, our study considered additional domains of well-being that have relationships with chronic pain, including other meaningful activities, social integration, and housing and physical environment.

### Strengths and Limitations

A major strength of this research study was the diverse range of participants in terms of geographical distribution and military service branches. Also, veterans reviewed the study instruments, which ensured that the questions were relevant and appropriate for the target population and the study’s aim. To increase reliability and trustworthiness, there were two coders for every interview transcript, and member-checking was conducted. Furthermore, our sample included a higher proportion of women (31%) compared to the proportion of women in the CAF (16%).^2^

There were some limitations of this study. The majority of participants came from a White or European background. Hence, the findings may not be generalizable to veterans of non-White racial backgrounds and may not have comprehensively covered the effect of racial background on veterans’ chronic pain experiences. Finally, the use of virtual or phone interviews may have excluded veterans who lacked reliable internet or phone access or were not comfortable using such technologies.

### Implications for Research and Practice

There were several implications for research derived from these findings. Various strategies such as social media, e-mail outreach, posters, and personal referrals were used to recruit participants; however, when these methods were endorsed by a fellow Veteran or a reputable Veteran organization, there was more success in participant recruitment. Future research wishing to explore the experiences of veterans may consider the importance and role of trust for this population and collaborate with existing organizations that support veterans for recruitment. A number of participants mentioned that their pain was not visible to others, which affected relationships with others, social integration, and social well-being. Further research could examine challenges and stigma related to “invisible” pain, injuries, or disabilities and their impact on veterans and access to care. Furthermore, we identified some unique challenges faced by women veterans in relation to chronic pain, including experiences of sexual trauma or harassment and their pain not being taking seriously, which was reinforced by a qualitive study of women veterans of the United States Armed Forces.^22^ Further research is needed to better understand the chronic pain experiences of women CAF veterans and barriers and facilitators in obtaining chronic pain services. Additionally, racial disparities in chronic pain care have been identified in the United States; for instance, Black veterans were less likely to visit pain clinics and more likely to visit the emergency department or urgent care for chronic pain compared to White veterans.^28^ Thus, future research should focus on the experiences of racialized VLwCP or those of other marginalized identities to identify ways to improve chronic pain care that addresses multiples aspects of vulnerability.

Furthermore, utilizing the domains of well-being to identify different aspects of well-being offered participants the opportunity to reflect on factors relating to their pain rather than prompting answers based on physical pain alone. Future research employing the domains of well-being may assist in understanding these factors holistically and develop an understanding of the relationships they may share. Future research can build on our findings about the experiences of VLwCP and relationships with the seven domains of well-being, as well as barriers and facilitators in accessing services, to determine whether these findings are representative among the larger population of VLwCP. For instance, a survey of Canadian veterans would be helpful to establish the generalizability of themes we discovered and to identify important targets for improving chronic pain care for VLwCP.

The findings from this study may improve the provision of care to veterans because it has elucidated barriers and facilitators that impact access to and interaction with healthcare and other support services. As some participants noted, the CAF and VAC have been changing their processes to address chronic pain, so some of these findings may reflect aspects of care that are already being addressed. Participants also noted the desire for a holistic approach to health. For this, HCPs may incorporate the use of the domains of well-being, as well as understandings of military culture and its impact on how veterans manage their chronic pain, to improve the treatment of chronic pain.

## Conclusion

Experiences of pain varied among Canadian veterans, and military culture played a role in their perceptions and management of pain. Barriers and facilitators to chronic pain care were highlighted from Veterans time in the military to their transition to civilian care. Participants described the impact of chronic pain on their overall well-being. Generalizability of these findings will be important for future research and knowledge translation to improve chronic pain care and quality of life for Canadian veterans.

## Supplementary Material

Supplemental Material

Supplemental Material

Supplemental Material
