# Supplementary material for: Canadian Veterans’ Experiences of Living with Chronic Pain: A Descriptive Qualitative Study
Source: Can J Pain. 2024 Jun 10;8(2):2361006. doi: 10.1080/24740527.2024.2361006 (PMC11382724; doi:10.1080/24740527.2024.2361006)
Supplement: Supplemental Material [file UCJP_A_2361006_SM0532.pdf]

Supplemental Material 3: Reflexive Exercise

Reporting of investigator reflexivity

**Research Team and Reflexivity** [a]	**Description**
Personal Characteristics	
Interviewer	
Credentials	
Occupation	
Gender	
Experience and training	
Relationship with Participants	
Relationship established	
Participant knowledge of the interviewer	
Interviewer characteristics	

[a]Adapted from the COnsolidated criteria for REporting Qualitative research (COREQ)
