# Supplementary material for: Canadian Veterans’ Experiences of Living with Chronic Pain: A Descriptive Qualitative Study
Source: Can J Pain. 2024 Jun 10;8(2):2361006. doi: 10.1080/24740527.2024.2361006 (PMC11382724; doi:10.1080/24740527.2024.2361006)
Supplement: Supplemental Material [file UCJP_A_2361006_SM0531.pdf]

Supplemental Material 2. Interview Guide

Semi-structured interview guide (45-60 minutes)

Participant ID______________________________________ Date______________________________

Location____ Researcher_________________________

Start time________________ End time________________ Date of informed consent ____________

Review and obtain informed consent - see informed consent form. Please note: (i) we will keep your information private, and will use only it for research, and (ii) you can stop me at any time. Do you agree to be interviewed? (Yes) or (No).

OK to record (circle): Y / N

-Thank you for agreeing to be part of this study. I will be asking you some in-depth questions about your experience as a Veteran living with chronic pain and use of pain services. Please answer as accurately as you can. Also, please feel free to ask me if you want any clarifications. This information will be kept confidential and any information used for data purposes will not link you to any of the information you provide. Have you fully reviewed the consent form? Do you have any questions before we start?-

<u>Semi-structured interview questions –</u>

1.1 Can you please tell me about your pain?
1.2 When did it start?
1.3 How did it start?
1.4 Did your pain start while you were in active duty?
1.5 How does your chronic pain affect your day-to-day work or tasks?
1.6 How does your chronic pain affect your relationships?
1.7 Do you feel that military culture affects your experience(s) of pain? If so, how?
1.8 Do you feel that military culture affects your management of pain? If so, how?
Probes – "mission first, team, regiment/unit, individual last", push through pain, team effort

2.1 What physical or mental health programs, supports, or services do you currently use for your chronic pain condition(s)?
2.2 Are these programs, supports, or services covered or provided by Veterans Affairs Canada?
Probes – physical (pain management, occupational therapy), psychological (psychotherapy, support groups, peer support) or pharmacological (medications) services

3.1 Have there been any barriers to you accessing services (physical, psychological or pharmacological) for chronic pain?

3.2 Have there been any concerns or barriers obtaining coverage from Veterans Affairs Canada (VAC) for chronic pain services, if applicable?
Probes – Navigating system and knowing what services are available, too much paperwork, services not covered, providers not accepting VAC, long wait times, feelings of keeping pain to oneself

4.1 Have there been any facilitators to you accessing services (physical, psychological or pharmacological) for chronic pain?
4.2 Have there been any facilitators to you obtaining coverage from Veterans Affairs Canada (VAC) for chronic pain services, if applicable?
Probes – Case manager, supportive family or friends, peer support (formal or informal), healthcare providers understanding of military experiences, services being nearby, accessible transportation to and from services, services covered, clear diagnosis and start date of diagnosis

5.1 Do you feel satisfied with the services you have received for chronic pain? And, why/why not?

6.1 Can you tell me about your transition from being in the military to using civilian or VAC services for chronic pain? What was the process like? Did you receive supports for this transition in care?
Probes – assignment and access to a case manager, access, service, cost, stigma

7.1 Veterans Affairs Canada (VAC) developed a framework for measuring well-being in Veterans that includes 7 domains – health, employment or other meaningful activity, finances, social integration, life skills, housing and physical environment, culture and social environment.

Please rate and describe how well you feel you are doing in each domain and what could be useful to improve in this domain (see Measuring Veteran Well-being handout for further description):

DOMAIN	Rate (0–well, no problem; 10-not well, many problems)	Do you feel this aspect of wellbeing has an effect on your chronic pain? If so, how?	Do you have supports / services/ programs to address this domain? If so, which ones	If you are receiving services / programs, are they through VAC? Are you satisfied with these services / programs? Why or why not?	Have your current treatment(s) for chronic pain considered this domain of wellbeing? If so, how?	What would you recommend could be useful to support you in doing well in this domain?
**Health –** A state of physical, mental. Social and spiritual functioning, broader than the absence of disease						
**Employment or other meaningful activity –** The sense of meaning attained by participating in fulfilling activities, such as employment						
**Finances –** Household income and financial security						
**Social integration –** Involvement in mutually supportive relationships (friends, family and community)						
**Life skills –** How personal health practices, coping skills and education enable management of life and contribute to resilience						

**Housing and physical environment –** Includes the built environment (housing), and the natural environment (water and air quality)						
**Culture and social environment –** The dominant values, beliefs, and attitudes of society that impact one's well-being						

8.1 Who else would be good to speak with to get a clear understanding of experiences of veterans living with chronic pain and access to care? Can you provide contact information for this/these individual(s)?
Probes – Other Veterans living with chronic pain, ways to engage other Veterans living with chronic pain

9.1 Is there any other information you feel I have left out which you would like to tell me regarding experiences of Veterans living with chronic pain or access to chronic pain care?

Thank you for your time.
Is it ok to contact you again if I need any clarifications or have other questions?
OK to contact for further interview: Y / N
Are you interested in reviewing and providing feedback on our preliminary findings (i.e., member checking) (Also see consent form)?
OK to contact for member checking: Y/N
You should be receiving your gift card via email within the next 2-5 business days. Thanks again!
