# Supplementary material for: Canadian Veterans’ Experiences of Living with Chronic Pain: A Descriptive Qualitative Study
Source: Can J Pain. 2024 Jun 10;8(2):2361006. doi: 10.1080/24740527.2024.2361006 (PMC11382724; doi:10.1080/24740527.2024.2361006)
Supplement: Supplemental Material [file UCJP_A_2361006_SM0530.pdf]

Supplemental Material 1. Demographic Questionnaire

Demographic questionnaire

Thank you for agreeing to be part of this study. Please answer the following questions as best as you can. There will be a chance to have questions explained if something is not clear. This information will be kept confidential and any information used for data purposes will not link you to any of the information you provide.

1. What is your age? (Select one)

☐ 18 to 25 years old
☐ 26 to 35 years old
☐ 36 to 45 years old
☐ 46 to 55 years old
☐ 56 to 65 years old
☐ 66 to 75 years old
☐ 76 years old and up

2. What best describes your current region of residence? (Select one)

☐ Urban (in the city)
☐ Rural (in the country)
☐ Suburban (mixed-use or residential area, existing either as part of a city area or as a separate residential community within commuting distance of a city)
☐ Other: ______________________________

3. In which Province or Territory do you currently live? (Select one)

☐ British Columbia
☐ Alberta
☐ Saskatchewan
☐ Manitoba
☐ Ontario
☐ Quebec
☐ New Brunswick
☐ Nova Scotia
☐ Prince Edward Island
☐ Newfoundland and Labrador
☐ Yukon
☐ Northwest Territories
☐ Nunavut
☐ Other ____

4. What was your sex assigned at birth? (Select one)

☐ Male
☐ Female

5. Which best describes your current gender identity? (Select one)

☐ Woman
☐ Man
☐ Transgender
☐ Gender neutral
☐ Non-binary
☐ Agender
☐ Pangender
☐ Genderqueer
☐ Two-spirit
☐ Other. I identify as: _______________

6. Which of the following best describes your race or ethnicity? (Select one)

☐ Asian – East (e.g. Chinese, Japanese, Korean)
☐ Asian – South (e.g. Indian, Pakistani, Sri Lankan, Indo-Caribbean/West Indian)
☐ Asian – Southeast (e.g. Malaysian, Filipino, Vietnamese, Cambodian)
☐ Asian – West (e.g. Afghani, Israeli, Saudi Arabian, Iranian, Turkish)
☐ Australasian – (e.g. Australia, New Zealand, New Guinea, Melanesia)
☐ Black – Africa (e.g. Ghanaian, Kenyan, Somali)
☐ Black – North America
☐ Black – Caribbean Region (e.g. Barbadian, Jamaican)
☐ Hispanic
☐ Indigenous (e.g. Inuit, First Nations, Non-Status Indian, Metis, Indigenous person from outside Canada)
☐ Latin American (e.g. Argentinean, Chilean, Salvadoran)
☐ White/European (e.g. English, Italian, Portuguese, Russian)
☐ Prefer not to answer
☐ Do not know
☐ Other: _____________________________

7. Primary language spoken (Select one)

☐ English
☐ French
☐ Indigenous language
☐ Other: ____

8. What best describes your current relationship status? (Select one)

☐ Single/Never Married
☐ Married
☐ Common law
☐ Separated
☐ Divorced
☐ Widowed

9. Who do you presently live with? (Check all that apply)

☐ Spouse/Partner
☐ Children
☐ Caregiver
☐ Grandchildren
☐ Parent(s)
☐ Roommate(s)
☐ Friend(s)
☐ No one, I live alone
☐ Other: ____

10. What best describes your current employment status? (Select one)

☐ Employed, full-time
☐ Employed, part-time
☐ Unemployed
☐ Unemployed, and receiving disability benefits
☐ Retired
☐ Other: _____________________________

11. What best describes your current household gross income level? (Select one)

☐ Below $25,000
☐ $25,000 to $49,999
☐ $50,000 to $74,999
☐ $75,000 to $99,999
☐ $100,000 to $150,000
☐ More than $150,000

12. What is your highest formal education level? (Select one)

☐ Elementary School
☐ High School
☐ College Degree
☐ University Degree
☐ Other: _____________________________

13. In which branch of the Canadian Armed Forces did you serve? (Select one)

☐ Army
☐ Navy
☐ Air Force
☐ Other: ____

14. For how long did you serve in the Canadian Armed Forces? (Select one)

☐ Less than 2 years
☐ 2-9 years
☐ 10-19 years

☐ >20 years

15. For how long have you been out of / retired from the Canadian Armed Forces? (Select one)

☐ Less than 2 years
☐ 2-9 years
☐ 10-19 years
☐ >20 years

16. For how long have you been living with chronic pain? (Select one)
Pain is defined by Health Canada as, "an unpleasant sensory or emotional experience associated with actual or potential tissue damage…Chronic pain is pain that continues for longer than 3 months." https://www.canada.ca/en/public-health/services/diseases/chronic-pain.html

☐ Less than 3 months
☐ 3 months – 6 months
☐ 6 months – 12 months
☐ 1-5 years
☐ 6-10 years
☐ More than 10 years

17. What type of chronic pain is it? (Select one)

☐ Neuropathic pain (pain caused by nerve injury or disease, such as sciatica or diabetic neuropathy)
☐ Nociceptive pain (pain caused by injury or disease affecting tissues other than nerves, such as osteoarthritis)
☐ Nociplastic pain (pain that results without a clearly defined injury, such as fibromyalgia or non-specific low back pain)
☐ Mixed type of pain (e.g. a combination of neuropathic, nociceptive and nociplastic pain)
☐ Do not know

18. How often do you have pain? (Select one)

☐ Daily
☐ Weekly
☐ Monthly
☐ Other ____

19. How bad is your chronic pain *on a regular basis* from 0 (no pain) to 10 (worst pain imaginable) (Place an x in the appropriate box)

0	1	2	3	4	5	6	7	8	9	10

No pain **Worst pain**

20. What pain management strategies are you *currently* using? (Check all that apply)

☐ Comprehensive pain program with a team of health care professionals
☐ Exercise therapy
☐ Stretching
☐ TENS: Transcutaneous electrical nerve stimulation
☐ Low level laser therapy
☐ Therapeutic ultrasound
☐ Pain education program
☐ Cognitive-behavioural therapy
☐ Family or group therapy
☐ Mind-body therapy (e.g., meditation)
☐ Spinal manipulation
☐ Natural or homeopathic remedies
☐ Acupuncture
☐ Massage therapy
☐ Over the counter medications: E.g., Tylenol, Advil
☐ Opioid medications: E.g., codeine, morphine, hydromorphone, tramadol
☐ Anti-depressants: E.g., duloxetine, amitriptyline
☐ Anti-seizure drugs: E.g., lamotrigine, gabapentin, carbamazepine
☐ Nonsteroidal anti-inflammatory drug (NSAID): E.g., naproxen, celecoxib
☐ Topical or dermal agents (applied to skin): E.g., capsaicin, lidocaine, topical diclofenac (Voltaren)
☐ Muscle relaxants: E.g., baclofen, tizanidine
☐ Cannabis
☐ Infusion of medications (e.g., lidocaine, ketamine)
☐ Nerve blocks and injections
☐ Nerve denervation (e.g., radiofrequency ablation)
☐ Others: ____

21. What pain management strategies have you *tried in the past*? (Check all that apply)

☐ Comprehensive pain program with a team of health care professionals
☐ Exercise therapy
☐ Stretching
☐ TENS: Transcutaneous electrical nerve stimulation
☐ Low level laser therapy
☐ Therapeutic ultrasound
☐ Pain education program
☐ Cognitive-behavioural therapy
☐ Family or group therapy
☐ Mind-body therapy (e.g., meditation)
☐ Spinal manipulation
☐ Natural or homeopathic remedies
☐ Acupuncture
☐ Massage therapy
☐ Over the counter medications: E.g., Tylenol, Advil
☐ Opioid medications: E.g., codeine, morphine, hydromorphone, tramadol

☐ Anti-depressants: E.g., duloxetine, amitriptyline
☐ Anti-seizure drugs: E.g., lamotrigine, gabapentin, carbamazepine
☐ Nonsteroidal anti-inflammatory drug (NSAID): E.g., naproxen, celecoxib
☐ Topical or dermal agents (applied to skin): E.g., capsaicin, lidocaine, topical diclofenac (Voltaren)
☐ Muscle relaxants: E.g., baclofen, tizanidine
☐ Cannabis
☐ Infusion of medications (e.g., lidocaine, ketamine)
☐ Nerve blocks and injections
☐ Nerve denervation (e.g., radiofrequency ablation)
☐ Others: ____

22. Do you have a chronic pain condition(s) approved by Veterans Affairs Canada (VAC) for which you are receiving services?

☐ Yes
☐ No

- *If no*, have you submitted a claim to Veterans Affairs Canada (VAC) for a chronic pain problem(s)?

☐ Yes
☐ No
